# Determinants of treatment response in first-episode psychosis: an ^18^F-DOPA PET study

**DOI:** 10.1038/s41380-018-0042-4

**Published:** 2018-04-20

**Authors:** Sameer Jauhar, Mattia Veronese, Matthew M Nour, Maria Rogdaki, Pamela Hathway, Federico E. Turkheimer, James Stone, Alice Egerton, Philip McGuire, Shitij Kapur, Oliver D Howes

**Affiliations:** 1 Department of Psychosis Studies, Institute of Psychiatry, Psychology and Neuroscience, King’s College, London, UK; 2 Early Intervention Psychosis Clinical Academic Group, South London & Maudsley NHS Trust, London, UK; 3 Centre for Neuroimaging Sciences, Institute of Psychiatry, Psychology and Neuroscience, King’s College, London, UK; 4 Psychiatric Imaging Group MRC London Institute of Medical Sciences, Hammersmith Hospital, London, W12 0NN UK; 5 Institute of Clinical Sciences, Faculty of Medicine, Imperial College, Hammersmith Hospital, London, W12 0NN UK; 6 Fiona Pepper, Centre for Neuroimaging Sciences, King’s College, London, UK

**Keywords:** Neuroscience, Biological techniques

## Abstract

Psychotic illnesses show variable responses to treatment. Determining the neurobiology underlying this is important for precision medicine and the development of better treatments. It has been proposed that dopaminergic differences underlie variation in response, with striatal dopamine synthesis capacity (DSC) elevated in responders and unaltered in non-responders. We therefore aimed to test this in a prospective cohort, with a nested case-control comparison. 40 volunteers (26 patients with first-episode psychosis and 14 controls) received an ^18^F-DOPA Positron Emission Tomography scan to measure DSC (Ki^cer^) prior to antipsychotic treatment. Clinical assessments (Positive and Negative Syndrome Scale, PANSS, and Global Assessment of Functioning, GAF) occurred at baseline and following antipsychotic treatment for a minimum of 4 weeks. Response was defined using improvement in PANSS Total score of >50%. Patients were followed up for at least 6 months, and remission criteria applied. There was a significant effect of group on Ki^cer^ in associative striatum (F_(2, 37)_ = 7.9, *p* = 0.001). Ki^cer^ was significantly higher in responders compared with non-responders (Cohen’s *d* = 1.55, *p* = 0.01) and controls (Cohen’s *d* = 1.31, *p* = 0.02). Ki^cer^ showed significant positive correlations with improvements in PANSS-positive (*r* = 0.64, *p* < 0.01), PANSS negative (rho = 0.51, *p* = 0.01), and PANSS total (rho = 0.63, *p* < 0.01) ratings and a negative relationship with change in GAF (*r* = −0.55, *p* < 0.01). Clinical response is related to baseline striatal dopaminergic function. Differences in dopaminergic function between responders and non-responders are present at first episode of psychosis, consistent with dopaminergic and non-dopaminergic sub-types in psychosis, and potentially indicating a neurochemical basis to stratify psychosis.

## Introduction

A significant proportion of people with schizophrenia and other psychotic disorders show poor response to antipsychotic treatment from illness onset [1, 2]. Poor therapeutic response is associated with worse functional outcomes, higher healthcare costs, and increased suicide risk [3]. Understanding the neurobiological basis of treatment response is fundamental to developing better pharmacological treatments. A meta-analysis of more than twenty molecular imaging studies showed both striatal dopamine synthesis capacity (DSC) and release are elevated in schizophrenia [4].This findings has also been seen in first-episode patients with affective, as well as schizophreniform psychoses [5-7]. Striatal DSC is also elevated in people at clinical high risk for psychosis [8, 9], and is specific to the later development of psychosis [8, 10], progressively increasing over the prodromal period [10], suggesting it is linked to development of psychosis. However, it has been proposed that there are at least two neurobiological sub-types underlying psychosis; a dopaminergic sub-type that shows good response to dopamine receptor antagonists, and a non-dopaminergic sub-type that shows poor response to dopamine receptor blockade [11]. Supporting this, cross-sectional imaging studies in chronic patients have found that patients with treatment-resistant schizophrenia do not show elevated striatal dopamine synthesis capacity relative to controls [12, 13]. Moreover, a post-mortem study of striatal levels of tyrosine hydroxylase, an enzyme involved in dopamine synthesis, found elevated levels in responders and no elevation in treatment non-responders [14]. Another study measured synaptic dopamine through indexing change in striatal dopamine D_2/3_ receptor radiotracer binding following pharmacological dopamine depletion in schizophrenia. It found the magnitude of alteration in radiotracer binding at baseline directly correlated with reduction in positive symptoms following antipsychotic treatment, indicating patients with lower synaptic dopamine levels show a poorer response to antipsychotics [15]. These studies suggest striatal DSC and baseline synaptic dopamine are linked to antipsychotic response.

Prior studies relating DSC to treatment response in psychosis included patients with chronic illness. As such it is unknown if differences in dopaminergic measures between antipsychotic responders and non-responders were present from illness onset, suggesting a ‘trait’ feature consistent with patient subgroups, or if differences had developed secondary to treatment effects or on-going symptoms, suggesting a “state” feature. We aimed to address these issues by prospectively examining the relationship between DSC and subsequent response to antipsychotic treatment in first-episode psychosis patients with minimal antipsychotic exposure. We focused on the associative striatum as the studies in chronic patients indicate that this is the main locus of dopaminergic alterations in psychosis [16], and for differentiating responders/non-responders [12]. We tested the following hypotheses:

1. Striatal dopamine synthesis capacity is higher in patients with psychosis whose illness responds to subsequent antipsychotic treatment, relative to non-responders and healthy controls, and unaltered in non-responders relative to controls.
2. Striatal dopamine synthesis capacity at presentation is positively associated with subsequent improvement in symptoms.

## Materials and methods

### Ethical approval

This study was approved by the East of England-Cambridge East NHS Research Ethics Committee, and Administration of Radioactive Substances Advisory Committee (ARSAC). All participants provided informed written consent to participate.

### Participants

Informed consent was obtained from all participants.

#### Inclusion criteria

Patients were recruited from clinical services for people presenting with a first episode of psychosis in South and West London. Inclusion criteria were: diagnosis of a psychotic disorder according to ICD 10 criteria [17], and requiring treatment with antipsychotic medication as determined by the treating clinician.

International Statistical Classification of Diseases and related health problems version 10 (ICD 10) criteria were used for diagnosis following entry into the study, at baseline and follow-up.

Healthy volunteers were recruited through local media from the same geographical area for normative comparisons. Inclusion criteria for controls were: no personal history of psychiatric illness (using the Structured Clinical Interview for the DSM) [18] and no concurrent psychotropic medication (ascertained through self-report).

Exclusion criteria for all subjects were: history of significant head trauma, dependence on illicit substances [19], medical co-morbidity (other than minor illnesses), lifetime use of antipsychotic drugs for longer than two weeks [20], contra-indications to PET and MRI scanning (such as pregnancy), or prescription of mood stabilizer medication.

### Medication status

Subjects with psychosis were classified by antipsychotic exposure as antipsychotic naïve, antipsychotic free (prior oral antipsychotic medication but free of treatment for at least 6 week (oral) or 6 months (depot, if relevant)) or minimally treated (taking antipsychotic medication for two weeks or less). Chlorpromazine-equivalent dose years were calculated for prior antipsychotic exposure (using the method described in [21]).

### Clinical assessment

All patients were clinically assessed at baseline, and reassessed after taking antipsychotic treatment at a therapeutic dose as specified in the Maudsley Prescribing Guidelines [19] for a minimum of four weeks, before determining response. Four weeks was chosen as the minimum duration of treatment based on evidence that most response to antipsychotic medication occurs within the first four weeks [22, 23], including in first-episode psychosis [24]. Furthermore, non-response before four weeks is a strong predictor of subsequent non-response [25].

All participants received follow-up for at least six months to determine if there had been a subsequent response in patients who showed non-response at four weeks. Choice of antipsychotic medication was determined by the treating clinician in discussion with the patient as per normal clinical care. Use of other psychotropic medication (e.g., antidepressants and benzodiazepines) was not an exclusion criterion for inclusion into the study; though additional psychotropic medication (antidepressant or mood stabilizer medication) during the study period (i.e., clinical follow-up) was an exclusion. Details of medication prescribed are shown in Supplementary Material.

Clinical measures were rated at baseline and follow-up using the Positive and Negative Syndrome Scale (PANSS) [26], Global Assessment of Functioning (GAF) [27], and Clinical Global Impression Improvement scale (CGI-I) [28]. Ratings were conducted by clinicians blinded to patients’ striatal DSC. The duration of illness was calculated from the onset of the first psychotic symptoms as previously described [29, 30].

### Determination of response and non-response

Our primary definition of response was a total PANSS reduction of ≥50% at the initial follow-up [31]. In line with recommendations to report treatment effects using more than one definition of response [31] we used two additional approaches to defining response.

The first used the CGI-Improvement (CGI-I) scale [28]. This scale rates global clinical improvement from 1 = ”Very much improved” to 7 = ”Very much worse”. A rating of 1 or 2 (corresponding to “very much improved” or “much improved”, respectively) corresponds to clinically significant improvement [32, 33]. The responder group was defined as a rating of 1 or 2 after treatment, corresponding to a clinically meaningful improvement, and the treatment non-responder group was defined as minimal improvement or worsening on the CGI-I. The second approach used the Andreasen et al. [34] remission criteria at 6 months to ascertain remission/ non-remission status.

### Medication concordance

To assess antipsychotic concordance, we used a multi-source approach, requiring evidence of adequate adherence on at least two of: antipsychotic levels in blood plasma, pharmacy and electronic medical dispensing records, reports from patients, and independent sources (a family member/carer or health care professional) [35]. Subjects were required to have taken at least 80% of prescribed doses, in line with recommendations [36].

To compare antipsychotic exposure after the scan we determined chlorpromazine-equivalent dose years (CPZ dose years), using the method described by Andreasen et al. [21]. In the case of drugs not covered by this (Lurasidone and Amisulpride) we used the approach described by Leucht et al. [37] and the Maudsley Prescribing Guidelines, respectively.

### ^18^F-DOPA PET imaging and analysis

Imaging data were obtained on a Siemens Biograph 6 HiRez PET scanner (Siemens, Erlangen, Germany) in three-dimensional mode. One hour before the scan participants received 400 mg entacapone, a peripheral catechol-*o*-methyl-transferase inhibitor, and 150 mg carbidopa, a peripheral aromatic acid decarboxylase inhibitor, to prevent formation of radiolabeled metabolites that may cross the blood–brain barrier [38]. Participants were positioned in the scanner with the orbitomeatal line parallel to the transaxial plane of the tomograph. Head position was marked, monitored and movement minimized using a head strap. After acquiring a CT scan for attenuation correction, ~150 MBq of ^18^F-DOPA was administered by bolus intravenous injection. PET data were acquired in 32 frames of increasing duration over the 95 min scan (frame intervals: 8 × 15 s, 3 × 60 s, 5 × 120 s, 16 × 300 s).

Region-of-interest (ROI) analysis was conducted blind to group status. Our primary endpoint was the striatal influx constant (Ki^cer^, written as K_i_ in previous publications [39]) in the associative striatum. SPM8 [40] was used to automatically normalize a tracer-specific template [41, 42] together with functional striatal ROI [43] and the reference region (the cerebellum) to each individual PET summation realigned image. Further details of striatal sub-divisions are given in Supplementary Material. Ki^cer^ was calculated using the Patlak-Gjedde graphical approach adapted for a reference tissue input function. This method has been shown to have good reliability for measuring Ki^cer^ (intra-class correlation coefficients >0.85 for striatal ROIs) [41]. An exploratory voxel-wise analysis was conducted to investigate sub-regional differences in Ki^cer^ between groups (Supplementary Material). Full details about tracer synthesis, data acquisition and analysis are given in Supplementary Material.

Individual striatal volumes were derived from the atlas based segmentation and calculated as the total volume of voxels in the striatal region-of-interest co-registered to the individual PET summed images (further details are given in Supplementary Material).

### Statistical Analysis

Statistical analyses were performed using SPSS, version 23 [44], and significance set to *p* < 0.05 (two-tailed). Normality of distribution was assessed using Shapiro–Wilkes test. To test the first hypothesis, we conducted a one-way analysis of variance (ANOVA) of the effect of group (responders, non-responders and control groups using the PANSS response criteria) on DSC, with post hoc pairwise Tukey tests to determine if Ki^*cer*^ in associative striatum was significantly different between groups, in line with our hypothesis that it is lower in non-responders relative to responders.

To test the second hypothesis, we examined the relationship between baseline associative striatal K_i_^*cer*^ and percentage improvement in positive psychotic symptoms (PANSS positive). Secondary analyses tested relationships between baseline K_i_^*cer*^ and improvement in other clinical measures, using Pearson’s correlation coefficients for normally distributed data, (PANSS positive) and Spearman’s correlation coefficients for non-normally distributed data (PANSS negative symptoms and GAF). Cook’s distance test was used to investigate potential outliers. ANOVA and independent sample t-tests were used to determine group differences for parametric demographic variables.

Percentage changes for GAF and PANSS were calculated, adjusting for minimum scores for the latter (7 for positive and negative symptom sub-scales, 30 for total symptoms) as shown here for the PANSS-positive symptom subscale:

1 $${\mathrm{\% }}\,\mathrm {change}\,\mathrm {in}\,\mathrm {positive}\,\mathrm {PANSS}\, = \,\frac{{\left( {\left( {\mathrm {baseline}\,\mathrm {score} - 7} \right) - \left( {\mathrm {follow}\,\mathrm {up}\,\mathrm {score} - 7} \right)} \right)}}{{(\mathrm {baseline}\,\mathrm {score} - 7)}} \cdot 100$$

Details of sample size calculation are given in Supplementary Material.

## Results

### Demographics

Demographic details of participants are given in Table 1.

**Table 1:** Demographic details

Variable	Total patient sample (*N*=26)	Responders (*N*=13)	Non-responders (*N*=13)	Controls (*N*=14)	*p*-value
Gender (M;F)	22;4	10;3	12;1	10;4	
Age, years^a^	25.31 (4.61)	24.38 (3.02)	26.23 (5.78)	24.29 (4.62)	*p* = 0.48
Ethnicity					
White	11	5	6	8	
Black	9	4	5	3	
Indian	2	2	0	2	
Other	4	2	2	1	
Duration of illness, months^b^	19 (25.5)	12.5 (20.3)	24 (35)	n/a	*p* = 0.08
Antipsychotic naive, *n* (%)/	14 (53.8%)/	9 (69.2%)/	5 (38.5)/		
Free, *n* (%)	9 (34.6%)	3 (23.1%)	6 (46.2%)		
Minimally treated, *n* (%)	3 (11.5%)	1 (7.7%)	2 (15.4%)		
Injected	146.02	144.62	147.41	146.92	*p* = 0.79
Activity, MBq^c^	(12.1)	(6.25)	(16.18)	(7.92)	
Smoking status					
Current	14 (53.8%)	9 (69.2%)	5 (38.5%)	3 (21.4%)	
smoker	4 (15.4%)	1 (7.7%)	3 (23.1%)	3 (21.4%)	
Past smoker	8 (30.8%)	3 (23.1%)	5 (385%)	8 (57.1%)	
Never smoker					

^a^Age is reported as mean (SD)

^b^Duration of illness is reported as Median (IQR)

^c^Injected Activity is reported as mean (SD)

Forty volunteers participated (26 patients, 14 healthy controls). There were no significant differences between groups (responders, non-responders and controls) in age, smoking status, radioactivity received, and between responders and non-responders in illness duration, and prior antipsychotic medication or antipsychotic exposure (measured in chlorpromazine dose years) between baseline scan and clinical follow-up (all *p*-values >0.05). ICD-10 diagnoses at baseline were: schizophrenia (*n* = 15), schizophreniform disorder (*n* = 1) and bipolar affective disorder (*n* = 10). At 6 month follow-up the only change in diagnosis was for the person with a diagnosis of schizophreniform disorder, which was changed to schizophrenia.

14 patients were antipsychotic naive, 9 medication-free, and 3 were minimally treated at time of scanning (further details in Supplementary Material). Of these, 13 subsequently met our primary (PANSS) criteria for treatment response, and 13 for non-response. Group composition varied little using the alternative response criteria (Supplementary Material).

### Baseline Ki^cer^ in treatment responders, non-responders and controls

There were no differences in baseline symptom severity between groups (Supplementary Table 1). There was no significant correlation between Ki^*cer*^ in associative striatum and baseline PANSS-positive, negative, total symptoms, or GAF score (all *p*-values >0.05).

**Fig. 1:**
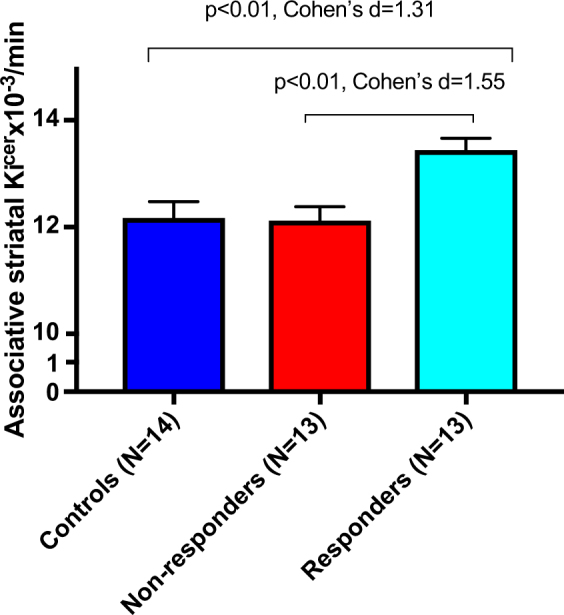
Mean dopamine synthesis capacity by group. Dopamine synthesis capacity is elevated at baseline in patients who subsequently respond to antipsychotic treatment, compared to controls and non-responders (Cohen’s *d* effect size = 1.31 and 1.55, respectively). Error bars indicate standard error of the mean

Fig. 1 shows dopamine synthesis capacity (DSC) by group. There was a significant effect of group on Ki^*cer*^ in the associative striatum (*F*_(2, 37)_ = 7.9, *p* = 0.001). Post hoc analyses, adjusted for multiple comparisons using the Tukey test, indicated Ki^*cer*^ was elevated in responders (mean = 13.45 × 10^−^^3^/min, SD = 0.78 × 10^−3^/min) relative to both non-responders (mean = 12.12 × 10^−3^/min, SD = 0.93 × 10^−3^/min, *p* = 0.004) and control groups (mean = 12.17 × 10^−3^/min, SD = 1.14 × 10^−3^/min, *p* = 0.004). The group effect remained significant after adjusting for cerebellar uptake (Supplementary Material).

Cohen’s d effect size for elevation in responders relative to non-responders was 1.55, and 1.31 relative to controls. Ki^cer^ values for whole striatum and functional sub-divisions are given in Supplementary Material. There were no statistically significant differences in Ki^cer^ between non-responders and controls (*p* = 0.99).

### Striatal volumes

There was no effect of group on striatal volume (*F*_(2, 37)_ = 2.22, *p* = 0.12), and pairwise comparisons also showed no significant difference in striatal volumes in responders (mean = 17.27 cm^3^, SD = 17.8 cm^3^) relative to non-responders (mean = 16.44 cm^3^, SD = 1.68 cm^3^, *p* = 0.38) and controls (mean = 17.71 cm^3^, SD = 1.26 cm^3^, *p* = 0.75), indicating that partial volume effects are unlikely to account for our group differences.

### Analysis in antipsychotic free individuals

In case antipsychotic treatment affected DSC, we conducted an exploratory analysis, excluding people taking antipsychotics at time of the scan, leaving 12 responders, 11 non-responders and 14 controls. There was a significant effect of group on Ki^*cer*^ in associative striatum (*F*_(2, 37)_ = 7.07, *p* = 0.001). Post hoc analyses, adjusted for multiple comparisons using Tukey test, indicated significant differences between responders (mean = 13.5 × 10^−3^/min, SD = 0.78 × 10^−3^/min), controls (*p* = 0.004), and non-responders (mean = 12.27 × 10^−3^/min, SD = 0.93 × 10^−3^/min, *p* = 0.01) and no significant difference between non-responders and controls (*p* = 0.96).

### Voxel-wise analysis

#### Responders vs non-responders

**Fig. 2:**
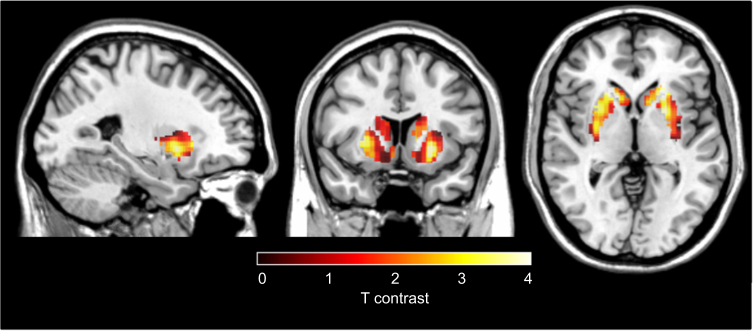
Voxel-wise analysis of treatment responders compared to non-responders. Increased dopamine synthesis capacity, relative to non-responder patients (*N* = 13), in patients who respond to treatment (*N* = 13). The most significant increase was in voxels in right caudate (Peak MNI coordinates *x* = 18, *y* = 20, *z *= 2; *p*_FWE corr_ = 0.026) and left putamen (Peak MNI coordinates *x* = −24, *y* = 8, *z* = −2; *p*_FWE corr_ = 0.027)

The voxel-based analysis identified greater K_i_^*cer*^ in responders relative to non-responders (defined using the PANSS criteria) in a voxel cluster with its peak in the left putamen, and right caudate, both within the associative striatum (Fig. 2, significant at *p* < 0.05 corrected for multiple comparisons using the family-wise error (FWE) rate method). The non-responder group>responder group contrast revealed no significant difference in any cluster or voxel, even at an uncorrected statistical threshold of *p* < 0.05.

#### Responders vs healthy controls

**Fig. 3:**
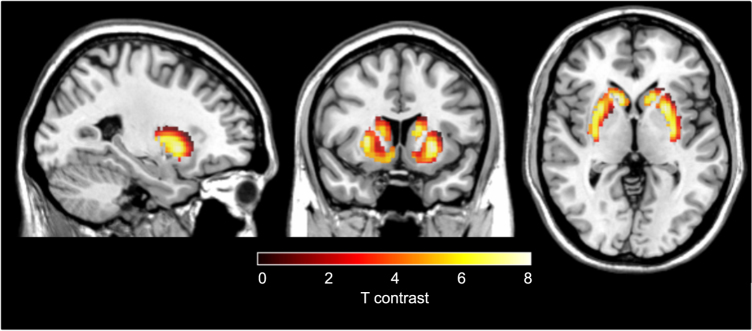
Voxel-wise analysis of treatment responders compared to controls. Increased dopamine synthesis capacity, relative to controls (*N* = 14), in patients who respond to treatment (*N* = 13). The most significant increase was in a voxel in both left (Peak MNI coordinates *x* = −12, *y* = 16, *z* = 6; *p*_FWE corr_ < 0.001) and right caudate (Peak MNI coordinates *x* = 14, *y* = 18, *z* = 0; *p*_FWE corr_ < 0.001)

The voxel-based analysis identified significantly greater K_i_^*cer*^ in responders relative to controls in both left and right caudate nuclei (Fig. 3, *p* < 0.05 FWE corrected for multiple comparisons). The control group>responder group contrast revealed no significant difference, even at an uncorrected statistical threshold of *p* < 0.05.

#### Non-responders vs healthy controls

The voxel-based analysis did not show any difference between non-responders and healthy controls for either non-responders>controls or controls>non-responders contrasts, even at an uncorrected statistical threshold of *p* < 0.05.

#### Secondary analyses using alternative criteria for response

One patient assigned to the non-responder group using the PANSS criteria for response, met the CGI criteria for response. Thus there were 14 responders and 12 non-responders in total using the CGI outcome measure. Nevertheless, using these groups, there was a significant effect of group status on Ki^cer^, (*F*_(2,37)_ = 6.05, *p* = 0.01). Ki^cer^ was significantly elevated in responders (mean = 13.32 × 10^−3^/min, SD = 0.88 × 10^-3^/min) relative to both non-responder (mean = 12.16 × 10^−3^/min, SD = 0.96 × 10^−3^/min, *p* = 0.01) and control groups (mean = 12.17 × 10^−3^/min, SD = 1.14 × 10^−3^/min, *p* = 0.02). There were no statistically significant differences in Ki^cer^ between non-responders and controls (*p* = 0.99). We also investigated whether baseline Ki^cer^ was associated with remission status at six months. There was a significant effect of Ki^cer^ on remission status at six months (*F*_(2, 37)_ = 6.05, *p* = 0.01). Ki^cer^ was significantly elevated in patients who subsequently met remission criteria (mean = 13.32 × 10^-3^/min, SD = 0.88 × 10^−3^/min) relative to those who did not meet remission criteria (mean = 12.16 × 10^−3^/min, SD = 0.96 × 10^−3^/min, *p* = 0.01).

### The relationship between baseline dopamine synthesis capacity and symptomatic and functional response

**Fig. 4:**
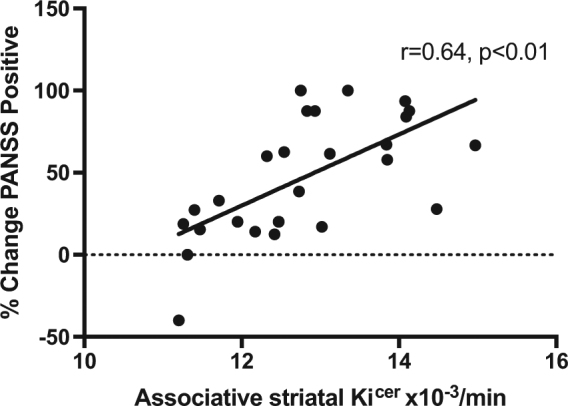
Relationship between baseline dopamine synthesis capacity and subsequent antipsychotic response. A positive association was found between associative striatum dopamine synthesis capacity and subsequent improvement in positive psychotic symptoms, measured using the PANSS-positive subscale

There was a significant positive correlation between associative striatal K_i_^cer^ and subsequent percentage improvement in PANSS-positive symptoms following treatment (*r* = 0.64, *p* < 0.001; Fig. 4). The relationship remained significant after removal of two potential outliers (*r* = 0.69, *p* < 0.01). The effect remained significant after partial correlation for dosage of antipsychotic medication received during treatment episode (CPZ dose years) (*r *= 0.44, *p* < 0.05). Whilst there was no significant difference in illness duration between responders and non-responders, non-responders had longer illness duration in absolute terms. To determine if this influenced the relationship with symptom response, we conducted a partial correlation including illness duration. The relationship between Ki^cer^ and positive symptoms remained significant after adjusting for illness duration (*r* = 0.66, *p* < 0.01).

Secondary analyses showed a significant positive correlation between baseline associative striatal K_i_^*cer*^ and percentage improvement in PANSS negative symptoms (rho = 0.51, *p* = 0.01), PANSS total symptoms (rho = 0.63, *p* < 0.01) and a significant negative correlation with GAF change (*r* = −0.55, *p* < 0.01). Ki^cer^ explained 41%, 26% and 40% of the variance for PANSS-positive, negative and total symptom change respectively, and 30% of variance in GAF improvement.

These correlations were numerically stronger when analysis was limited to antipsychotic naïve subjects for percentage change in PANSS-positive symptoms (*r* = 0.77; *p* < 0.01), for percentage change in PANSS negative symptom (rho = 0.65; *p* = 0.01), for percentage change in total PANSS symptoms (rho = 0.76; *p* < 0.01), and for GAF improvement (*r* = −0.79; *p* < 0.01).

## Discussion

Our main finding is that striatal dopamine synthesis capacity is significantly higher in patients with first-episode psychosis who subsequently respond to antipsychotic treatment compared to those with a subsequent poor response, with a large effect size (Cohen’s d = 1.55). Striatal dopamine synthesis capacity explained >40% of the variance in subsequent positive psychotic symptom change following treatment. This suggests dopamine dysfunction before starting treatment is linked to likelihood of responding to antipsychotic treatment, such that those with greatest dopamine elevation during an acute psychotic episode are most likely to show symptomatic improvement during treatment.

This extends a prior cross-sectional study showing elevated DSC in chronic treatment-responsive patients relative to non-responders [13], to show prospectively that dopamine function is linked to response in first-episode patients. Our findings suggest differences between responders and non-responders are not secondary to illness chronicity or long-term antipsychotic exposure. Furthermore, the effect size for the differences that we identified was similar to patients with chronic psychoses, suggesting the magnitude of differences may not change markedly over illness course or with long-term medication exposure, although longitudinal studies are required to definitively test this.

These findings also extend other recent evidence that response to antipsychotic treatment in first-episode patients is related to alterations in striatal functional connectivity [45–47], and lower baseline striatal D_2/3_ receptor availability between responders and non-responders [48]. It has been speculated that lower baseline D_2/3_ receptor availability could reflect greater synaptic dopamine levels in responders [48], which would be consistent with our finding that responders also have elevated dopamine synthesis capacity. However, people with established schizophrenia (both responsive and resistant to treatment) show no differences in D_2/3_ receptor availability compared with healthy controls [49]. Taken with previous molecular imaging studies showing dopamine D_2/3_ receptor occupancy by antipsychotics is required for therapeutic response [33, 50], our findings indicate D_2/3_ receptor blockade is acting to oppose the consequences of presynaptic dopamine dysfunction [51].

### Strengths and limitations

A strength of this study is its prospective design in first-episode patients predominantly free of antipsychotic medication. Our measure for defining response has good clinical validity [52], and was reinforced by finding similar results using other outcomes. A potential limitation is that patients received a range of different antipsychotics. However, both responder and non-responders received similar antipsychotics (Supplementary Material), and there is good evidence that these compounds have similar effectiveness [24]. Furthermore, partial correlation for effects of antipsychotic exposure on the correlation between Ki^*cer*^ and PANSS-positive symptom improvement did not change our results, making it unlikely that the differences in response were due to differences in treatment exposure. Three of 26 subjects (one responder and two non-responders) had received a short course of antipsychotic treatment prior to imaging, which could have conceivably affected DSC. The preclinical animal literature suggests that, by blocking D_2_ autoreceptors, acute antipsychotic use would increase presynaptic dopamine synthesis [53, 54], but chronic use leads to reduced dopamine neuron firing, which could be expected to reduce dopamine synthesis [53]. The acute effects of antipsychotics on DSC in healthy volunteers are inconsistent [55, 56]. The only longitudinal study in patients showed a reduction in striatal dopamine synthesis after approximately 5 weeks of haloperidol treatment [57]. Overall it remains unclear what effect the treatment these patients had received would have had on dopamine synthesis capacity. In any event, our findings remained significant when group analyses excluded minimally treated patients, and when correlation analyses were restricted to antipsychotic naive subjects, indicating that this does not account for the alterations we report. Our study design did not enable us to investigate the relationship between DSC and the development of treatment resistance, which would require non-responders to be switched to a second antipsychotic.

Our study was in patients presenting with their first psychotic episode, and included patients with diagnoses of schizophrenia and bipolar affective disorder, in line with other first-episode studies [46, 7]. As such our results relate to psychosis across these disorder, specifically positive psychotic symptoms. Nevertheless, it is important to recognize that diagnoses can change over the first few years of illness [58] and further follow-up of our sample is required to determine the final diagnoses and if findings are specific to particular psychotic disorders.

Finally, we did not measure the plasma input function so cannot exclude differences in peripheral ^18^F-DOPA metabolism or other factors affecting delivery of ^18^F-DOPA to brain tissue. However, we blocked peripheral metabolism and previous studies have not detected differences in psychotic disorders [59, 60]. Differences in blood flow could also influence findings. However, the reference region approach we used accounts for global differences in ^18^F-DOPA delivery [60], and co-varying for tracer uptake in the cerebellum (SUV) did not change the results.

### Biological meaning of our findings

Ki^cer^ indexes a number of biological processes in vivo, including the uptake of radiolabeled DOPA into dopamine neurons, its conversion into radiolabeled dopamine by DOPA decarboxylase, and its storage in vesicles in presynaptic dopamine nerve terminals [54]. As such it does not necessarily imply increased dopamine synthesis, as the measure is also sensitive to other processes such as L-DOPA transport into the neuron, vesicular storage, and dopamine catabolism. Moreover, the rate-limiting step for dopamine synthesis in brain is the conversion of tyrosine into L-DOPA by tyrosine hydroxylase. Nevertheless, DOPA decarboxylase is a regulated enzyme in the synthesis of dopamine, may be rate limiting in some circumstances, and dopamine levels are sensitive to DOPA decarboxylase activity [7, 60]. Moreover, studies using challenge paradigms indicate that patients with psychotic disorders show more striatal dopamine release than healthy controls [61, 62], dopamine turnover is elevated in schizophrenia [63] and greater synaptic dopamine levels are linked to greater subsequent improvement in psychotic symptoms [15]. Thus, taken with this prior evidence, the most parsimonious explanation of our findings is that increased dopamine synthesis capacity is linked to subsequent treatment response in psychotic disorders (see Howes et al. [4] for a further discussion). Nevertheless, future studies of tyrosine hydroxylase activity in patients would be useful to determine if this is altered in vivo and provide further evidence that dopamine synthesis is related to treatment response.

### Implications and Future directions

Our findings that antipsychotic treatment response in psychosis is related to pre-existing presynaptic dopamine function provides an explanation for the effectiveness of antipsychotics, which antagonize dopamine receptors [64], and their ineffectiveness in the non-responders, who did not show marked dopaminergic dysfunction as a group. Our findings suggest novel treatments with a mechanism of action that does not involve dopaminergic blockade may be indicated in this subgroup. These findings also suggest neuroimaging measures, in addition to clinical variables, could be used to help stratify patients with psychosis to predict future antipsychotic response and, more importantly, non-response. This could facilitate earlier introduction of alternatives to conventional treatment, such as clozapine, or novel interventions that do not depend on dopamine receptor antagonism.

Further work is required to determine the accuracy of F-DOPA PET in predicting response, and whether this is sufficient on its own or additional clinical measures are required. While ^18^-F-DOPA PET is not widely available, limiting its current use as a biomarker, the increasing availability of PET scanners and the relatively long half-life of the ligand, allowing it to be manufactured centrally and delivered to many sites, means this is likely to change in the next few years.

In conclusion, striatal dopamine synthesis capacity is elevated in patients with first-episode psychosis who subsequently respond to treatment, but not in those who do not respond, and the level of dopamine synthesis capacity is associated with degree of subsequent antipsychotic response.

## Electronic supplementary material

Supplemenmtary Material Unmarked(DOCX 43 kb)
