# Supplementary material for: Determinants of treatment response in first-episode psychosis: an 18F-DOPA PET study
Source: Mol Psychiatry. 2018 Apr 20;24(10):1502–12. doi: 10.1038/s41380-018-0042-4 (PMC6331038; doi:10.1038/s41380-018-0042-4)
Supplement: Supplementary file 1 — Supplemenmtary Material Unmarked(DOCX 43 kb) [file 41380_2018_42_MOESM1_ESM.docx]

**Determinants of antipsychotic treatment response in first episode psychosis: an ^18^F-DOPA PET study**

**Jauhar S. *et al***

**SUPPLEMENTARY MATERIAL**

**Sample size calculation**

We used data from a prior study in schizophrenia, which showed an effect size of 1.3 for the elevation in associative striatal dopamine synthesis capacity between treatment responders and treatment resistant populations^1^, to inform the power calculation. This determined that a sample size of at least 11 would have greater than 80% power to detect a difference between groups, and at least 21 to detect a moderate or greater correlation with symptoms both with an alpha value of <0.05 (two tailed).

^18^F-DOPA PET imaging

All participants were asked not to eat or drink (except water), and refrain from alcohol for 12 hours prior to scanning. Cigarette smokers were not permitted to smoke for four hours preceding the scan ^2^.

^18^F-DOPA synthesis and PET data acquisition

A 17MeV GE PET-trace cyclotron was used for radionuclide production. The gas target was filled with ^18^O_2_ and bombarded at 40 mA for 30 min followed by a passivation bombardment of 0.1% F_2_ in argon at 20 mA for 20 min. This produced ^18^F-F by the ^18^O (p,n) ^18^F reaction. An electrophilic fluorination procedure was then used to synthesize 6-^18^F fluoro-L-DOPA. In brief, [^18^F]F2 was bubbled through a solution of 6-trimethylstannyl-L-DOPA (60 mg) stirring in Deutero-chloroform (5 ml) over 20 mins at 5 1C. 6 M HCl (2 ml) was added and the chloroform evaporated at 70 1C. The resulting aqueous mixture was heated at reflux for 10 mins before allowing to cool. The cooled crude mixture was purified by semi-prep high-pressure liquid chromatography polymer X column eluting with ammonium acetate buffer. The peak corresponding to ^18^F-L-DOPA eluted at 15 mins was stabilized with 1 mg ascorbic acid and sodium phosphate dibasic. For quality assurance purposes, a sample was taken from each synthesis and analyzed by reverse phase high-pressure liquid chromatography to confirm identity and purity. To proceed with the injection, a radiochemical purity of 95.0% or higher was required.

***PET data analysis***

Correction for head movement during scan was performed by denoising the non-attenuation-corrected dynamic images using a level 2, order 64 Battle-Lemarie wavelet filter.  Frames were realigned to a single reference frame, acquired 20 mins post-injection, employing a mutual information algorithm ^3,4^. The transformation parameters were then applied to the corresponding attenuated-corrected dynamic images, creating a movement-corrected dynamic image, which was used in the analysis. Realigned frames were then summated to create an individual motion-corrected reference map for the brain tissue segmentation. SPM8^5^ was used to normalize a tracer-specific (^18^F-DOPA) template ^6,7^ together with the a striatal probabilistic brain atlas defined by Martinez et al ^8^ both in the same space to each individual PET summation image. The brain atlas was used to identify the whole striatum, functional striatal subdivisions and the cerebellar region used as reference for tissue quantification ^8^. The striatal influx constant (Ki^cer^, written as K_i_ in some previous publications ^7^) was calculated compared with uptake in the reference region using the Patlak-Gjedde graphical approach adapted for a reference tissue input function ^6^. A previous test/re-test study has shown this approach has good reliability ^6^. Further details of the image analysis approach are given in Bloomfield et al^2^.

Though the reference region approach is robust to global differences in radiotracer delivery to the brain^9^, we examined the reference region (cerebellum) to see whether there was any change in standardized uptake value (SUV) in the cerebellum at 95 minutes.

Striatal volume measures were derived from the atlas based segmentation as the number of voxels in the striatal region times the volume of a single PET image voxel (voxel volume = 2.05mm x 2.05mm x 2mm = 8.41mm^3^). This analysis was undertaken to investigate the possible presence of partial volume differences between groups.

PET parametric mapping

We implemented a previously established method^10^ in which K_i_^cer^ parametric images of the brain were constructed from motion-corrected images using a wavelet-based approach^11^. The parametric image for each participant was then normalized into Montreal Neurological Institute standard space (matrix dimension: 91x109x91; voxel size: 2mm isotropic) using the participant’s PET summation image and the ^18^F-DOPA template. Statistical parametric mapping was conducted using SPM8 using a striatal mask^10^ to compare striatal dopamine synthesis capacity between responders and non-responders using an independent t-test. Results are presented corrected for multiple comparisons as applied in SPM8 (family wise error rate (FWE) corrected).

**SUPPLEMENTARY TABLES**

**Supplementary Table 1 Clinical variables in patient sample**

| **Clinical**  **variable** | **Total sample** | | **Responders** | | **Non-responders** | |
| --- | --- | --- | --- | --- | --- | --- |
|  | **Baseline** | **Follow-up** | **Baseline** | **Follow-up** | **Baseline** | **Follow-up** |
| **PANSS Positive** | 19.96  (6.04) | 14.12  (5.89) | 20.69  (7.62) | 10.00  (2.45) | 19.23  (4.09) | 18.23  (5.43) |
| **PANSS Negative** | 16.81  (5.82) | 13.5  (6.04) | 17  (5.51) | 9.77  (2.77) | 16.62  (6.33) | 17.23  (6.18) |
| **PANSS Total** | 73.65 (6.04) | 54.22  (18.92) | 74.23 (16.96) | 42.15  (7.61) | 73.08  (14.63) | 72.23 (18.72) |
| **GAF** | 47.85 (14.36) | 66.3  (16.68) | 47.77  (10.86) | 77.31  (9.04) | 47.92  (17.65) | 55.31 (15.34) |

|  | **Responders**  **(N=13)** | **Non-responders**  **(N=13)** |
| --- | --- | --- |
| **Medication** | Amisulpride, N=5  Aripiprazole, N=2  Olanzapine, N=1  Quetiapine, N=2  Risperidone, N=1  Lurasidone, N=1  Amisulpride and Aripiprazole N=1 | Amisulpride, N=1  Aripiprazole, N=1  Olanzapine, N=4  Quetiapine, N=1  Quetiapine and Sertraline, N=1  Amisulpride and Quetiapine N=1  Risperidone and Aripiprazole, N=1  Risperidone and Mirtazapine, N=1  Paliperidone N=1  Aripiprazole depot N=1 |
| **Chlorpromazine equivalent (dose years) prior to scan**  **(Median, IQR)** | 0 (0.1) | 0 (0.3) p=0.46 |
| **Chlorpromazine equivalents (dose years) between scan and follow-up clinical assessment** | 0.38 (0.29) | 0.53 (0.35) p=0.26 |

**Supplementary Table 2 Psychotropic medication**

At six-month follow-up, all PANSS response criteria responders met criteria for remission, ands 1 of the 13 PANSS criteria non-responders fulfilled criteria for remission.

All CGI-I responders met remission criteria, whilst all CGI-I non-responders continued to meet the non-remission criteria.

**Supplementary Table 3**

**Response and remission criteria applied to patient sample**

| **CGI-Improvement score** | **Total (n)** | **Very much worse** | **Much worse** | **Minimally worse** | | **Unchanged** | **Minimally better** | **Much better** | **Very much better** |
| --- | --- | --- | --- | --- | --- | --- | --- | --- | --- |
| **n** | **26** |  | **1** | **3** | **6** | | **2** | **9** | **5** |
|  |  |  |  |  |  | |  |  |  |
| **PANSS Total reduction** | **Total (n)** | **<=0% PANSS reduction** | **0-<25% PANSS reduction** | **25-49% PANSS reduction** | **50-74% PANSS reduction** | | **75-100% PANSS reduction** |  |  |
|  | 26 | 3 | 8 | 2 | 7 | | 6 |  |  |

**Supplementary Table 4 Dopamine synthesis capacity in striatal sub-regions**

| **Striatal region** | **Mean (sd) Ki^cer^ Responder**  **(n=13)** | **Mean (sd) Ki^cer^ Non-responder**  **(n=13)** | **Mean(sd)Ki^cer^ Controls**  **(n=14)** | |
| --- | --- | --- | --- | --- |
| **Whole striatum**  **(sig at p=0.01)** | 13.38 x 10^-3^  (0.74 x 10^-3^) | 12.27 x 10^-3^  (0.92 x 10^-3)^ | 12.26 x 10^-3^  (1.21 x 10^-3)^ |  |
| **Associative Striatum** | 13.45x10^-3^  (0.78 x 10^-3^) | 12.12 x 10 ^-3^  (0.93x10^-3^) | 12.17 x 10^-3^  (1.14 x 10^-3^) |  |
| **Limbic Striatum** | 12.98 x 10^-3^ (0.76 x 10^-3^) | 12.35 x 10^-3^  (0.87 x 10^-3^) | 12.14 x 10^-3^ ( (1.25 x 10^-3^) |  |
| **Sensorimotor Striatum** | 13.41 x 10^-3^ (0.92x 10^-3^) | 12.6 x 10^-3^  (1.1 x 10^-3^) | 12.53 x 10^-3^ (1.44 x 10^-3^ |  |

**SUV analysis**

We conducted an ANCOVA, with SUV in the reference region as a covariate, to see if tracer uptake in the cerebellum may account for our results, using the reference region (cerebellum) approach. This did not change the results, F (2,56)=5.74, p=0.01.

**References**

1 Demjaha A, Murray RM, McGuire PK, Kapur S, Howes OD. Dopamine synthesis capacity in patients with treatment-resistant schizophrenia. *American Journal of Psychiatry* 2014.http://ajp.psychiatryonline.org/doi/10.1176/appi.ajp.2012.12010144 (accessed 19 Dec2015).

2 Bloomfield MAP, Pepper F, Egerton A, Demjaha A, Tomasi G, Mouchlianitis E *et al.* Dopamine function in cigarette smokers: an [^18^F]-DOPA PET study. *Neuropsychopharmacology* 2014; **39**: 2397–2404.

3 Studholme C, Hill DL, Hawkes DJ. Automated 3-D registration of MR and CT images of the head. *Med Image Anal* 1996; **1**: 163–175.

4 Turkheimer FE, Brett M, Visvikis D, Cunningham VJ. Multiresolution analysis of emission tomography images in the wavelet domain. *J Cereb Blood Flow Metab* 1999; **19**: 1189–1208.

5 SPM - Statistical Parametric Mapping. http://www.fil.ion.ucl.ac.uk/spm/ (accessed 5 Oct2016).

6 Egerton A, Demjaha A, McGuire P, Mehta MA, Howes OD. The test-retest reliability of 18F-DOPA PET in assessing striatal and extrastriatal presynaptic dopaminergic function. *Neuroimage* 2010; **50**: 524–531.

7 Howes OD, Montgomery AJ, Asselin MC, Murray RM, Valli I, Tabraham P *et al.* Elevated striatal dopamine function linked to prodromal signs of schizophrenia. *Archives of general psychiatry* 2009; **66**: 13.

8 Martinez D, Slifstein M, Broft A, Mawlawi O, Hwang D-R, Huang Y *et al.* Imaging Human Mesolimbic Dopamine Transmission With Positron Emission Tomography. Part II: Amphetamine-Induced Dopamine Release in the Functional Subdivisions of the Striatum. *J Cereb Blood Flow Metab* 2003; **23**: 285–300.

9 Kumakura Y, Cumming P. PET studies of cerebral levodopa metabolism: a review of clinical findings and modeling approaches. *The Neuroscientist* 2009.http://nro.sagepub.com/content/early/2009/09/29/1073858409338217.short (accessed 9 Mar2015).

10 Howes OD, Bose SK, Turkheimer F, Valli I, Egerton A, Valmaggia LR *et al.* Dopamine synthesis capacity before onset of psychosis: a prospective [18F]-DOPA PET imaging study. *Am J Psychiatry* 2011; **168**: 1311–1317.

11 Turkheimer FE, Aston JAD, Asselin M-C, Hinz R. Multi-resolution Bayesian regression in PET dynamic studies using wavelets. *NeuroImage* 2006; **32**: 111–121.
